# Effects of Accumulated Energy on Nanoparticle Formation in Pulsed-Laser Dewetting of AgCu Thin Films

**DOI:** 10.1186/s11671-021-03564-5

**Published:** 2021-06-30

**Authors:** H. K. Lin, C. W. Huang, Y. H. Lin, W. S. Chuang, J. C. Huang

**Affiliations:** 1 Graduate Institute of Materials Engineering, National Pingtung University of Science and Technology, 1, Hseuhfu Road, Pingtung 912, Taiwan, ROC; 2 Department of Plant Medicine, National Pingtung University of Science and Technology, 1, Hseuhfu Road, Pingtung 912, Taiwan, ROC; 3 Department of Materials Science and Engineering, Hong Kong Institute for Advanced Study, City University of Hong Kong, Kowloon, Hong Kong

**Keywords:** Pulsed-laser dewetting, Double-peak absorption spectrum, Surface plasmon resonance, AgCu eutectic system

## Abstract

Ag_50_Cu_50_ films were deposited on glass substrates by a sputtering system. Effects of accumulated energy on nanoparticle formation in pulse-laser dewetting of AgCu films were investigated. The results showed that the properties of the dewetted films were found to be dependent on the magnitude of the energy accumulated in the film. For a low energy accumulation, the two distinct nanoparticles had rice-shaped/Ag_60_Cu_40_ and hemispherical/Ag_80_Cu_20_. Moreover, the absorption spectra contained two peaks at 700 nm and 500 nm, respectively. By contrast, for a high energy accumulation, the nanoparticles had a consistent composition of Ag_60_Cu_40_, a mean diameter of 100 nm and a peak absorption wavelength of 550 nm. Overall, the results suggest that a higher Ag content of the induced nanoparticles causes a blue shift of the absorption spectrum, while a smaller particle size induces a red shift.

## Introduction

Noble metallic nanoparticles have been widely researched due to their many interesting physical characteristics; interesting electrochemical and mechanical properties [1–3]. One of the most important properties of such nanoparticles is their localized surface plasmon resonance (LSPR), which originates from the interaction between the incident light and the free electrons on the metallic surface [4]. In particular, the time-varying electric field associated with the incident light exerts a force on the free electrons, which causes them to oscillate [5]. At a certain excitation frequency, the oscillation of the surface free electrons coincides with that of the incident light and the resulting resonance leads to a significant increase in the light absorption of the surface at the corresponding wavelength. Metallic nanoparticles exhibit a localized surface plasmon resonance behavior when their size reduces to a scale less than that of the wavelength of the incident light [6].

Among the various metallic materials in common use, silver (Ag) and copper (Cu) have been extensively explored and have found widespread use in the antibacterial [7, 8], photovoltaic [9, 10], optoelectronic [9, 11], and electrocatalysis [12] fields. In many such applications, it is desirable to pattern metallic nanoparticles on the substrate surface. This is commonly performed using a laser dewetting process [13–16]. The literature contains many studies on the formation of metallic nanoparticles through laser dewetting [17]. However, most of these studies focus on the dewetting of pure metals [14, 16–18]. In other words, the literature contains only scant information on the laser dewetting of alloys [13, 15]. However, thin alloy films with nanoparticle structures are of great practical importance in many applications, including surface plasmon resonance and optical hydrogen sensors [19, 20]. Ruffino reported that the absorbance of the surface structures in arrays fabricated at various periods would clearly show the possibility to tune the plasmonic properties by tuning the arrays’ geometrical characteristics [17]. The sensing property of the metallic nanoparticles owing to their characteristic surface plasmon resonance (SPR) is under development [21]. The sensing ability of the synthesized nanoparticles was further supported by Raman spectroscopy. The synthesized nanoparticles were further employed for the sensing of pesticide using absorption spectral technique [22]. Thus, further research into the effects of laser dewetting on the chemical and mechanical properties of thin alloy films is required.

The properties of pure Ag and Cu under laser dewetting are well understood [13, 23]. Despite many assorted applications of monometallic nanoparticles, the synthesis of bimetallic nanoparticles has also accelerated due to the combined properties of the constituent metals. For example, the bimetallic nanoparticles have enhanced reactivity over their monometallic counterparts in the catalysis field [24]. Thus, in seeking to clarify the dewetting mechanism in alloy systems, the present study deliberately chooses AgCu alloy as the research target. In particular, equimolar AgCu thin films are deposited on glass substrates and the morphologies, compositions and absorption properties of the films are examined following laser dewetting performed with different laser pulse repetition rates, laser powers and scanning speeds.

## Methods

Ag_50_Cu_50_ films with a thickness of 10 nm were co-sputtered from pure Ag and Cu targets on glass substrates (Nippon Electric Glass Co., thickness: 7 mm, surface roughness: 1.8 nm) using a high-vacuum sputtering system with a base pressure of 2 × 10^−6^ torr and an Ar gas flow rate of 30 sccm. The microstructures of the as-deposited Ag_50_Cu_50_ films (100 nm) were examined using a D8 X-ray Diffractometer (XRD, Bruker D8 Advance) with Cu-Kα radiation (*λ* = 0.1540 nm) and an operating voltage and current of 40 kV and 30 mA, respectively. The as-deposited films (10 nm) were then dewetted using a pulsed near-infrared radiation (NIR) laser system (SPI-12, UK Fiber Laser) with a wavelength of 1064 nm, a pulse duration of 200 ns and a spot size with 40 μm. To investigate the effect of different processing conditions on the nanoparticle formation in the thin films, the dewetting process was performed using two repetition rates (100 and 300 kHz), four pulse powers (2, 6, 8 and 12 W) and four scanning speeds (50, 400, 800 and 1200 mm/s). In every case, the scan pitch was set as 20 μm. For each dewetting process, the pulse energy (E) was calculated as [25]:

1 $$E = P_{{{\text{AVG}}}} {\text{/rep}},$$

where *P*_AVG_ is the average power of the laser and rep is the repetition rate. For the processing conditions considered in the present study, the pulse energy ranged from 6.7 to 120 μJ.

The optical properties of the dewetted samples were analyzed using a UV–vis-IR spectrophotometer (Lambda 35, PerkinElmer) at wavelengths ranging from 300 to 1000 nm. The surface morphologies of the dewetted samples were observed by a Field-Emission Scanning Electron Microscope (FE-SEM, JSM-7600F). The particle size distribution was measured using ImageJ image-processing software (National Institutes of Health, USA) with a minimum of 100 particles per sample. Finally, the microstructures and element compositions of the as-deposited film and nanoparticles were examined using a Field-Emission Transmission Electron Microscope (FE-TEM, Tecnai F20 G2) equipped with Energy Dispersive X-ray Spectrometry (EDS). To fabricate the TEM samples, an ultra-thin layer of Pt was deposited on the sample surface in order to protect the nanoparticles during milling. Then, a Focus Ion Beam system (FIB, Hitachi NX2000) was then used to accurately cut and mill the cross-section of the chosen dewetted nanoparticles into TEM samples.

## Results and Discussions

**Fig. 1:**
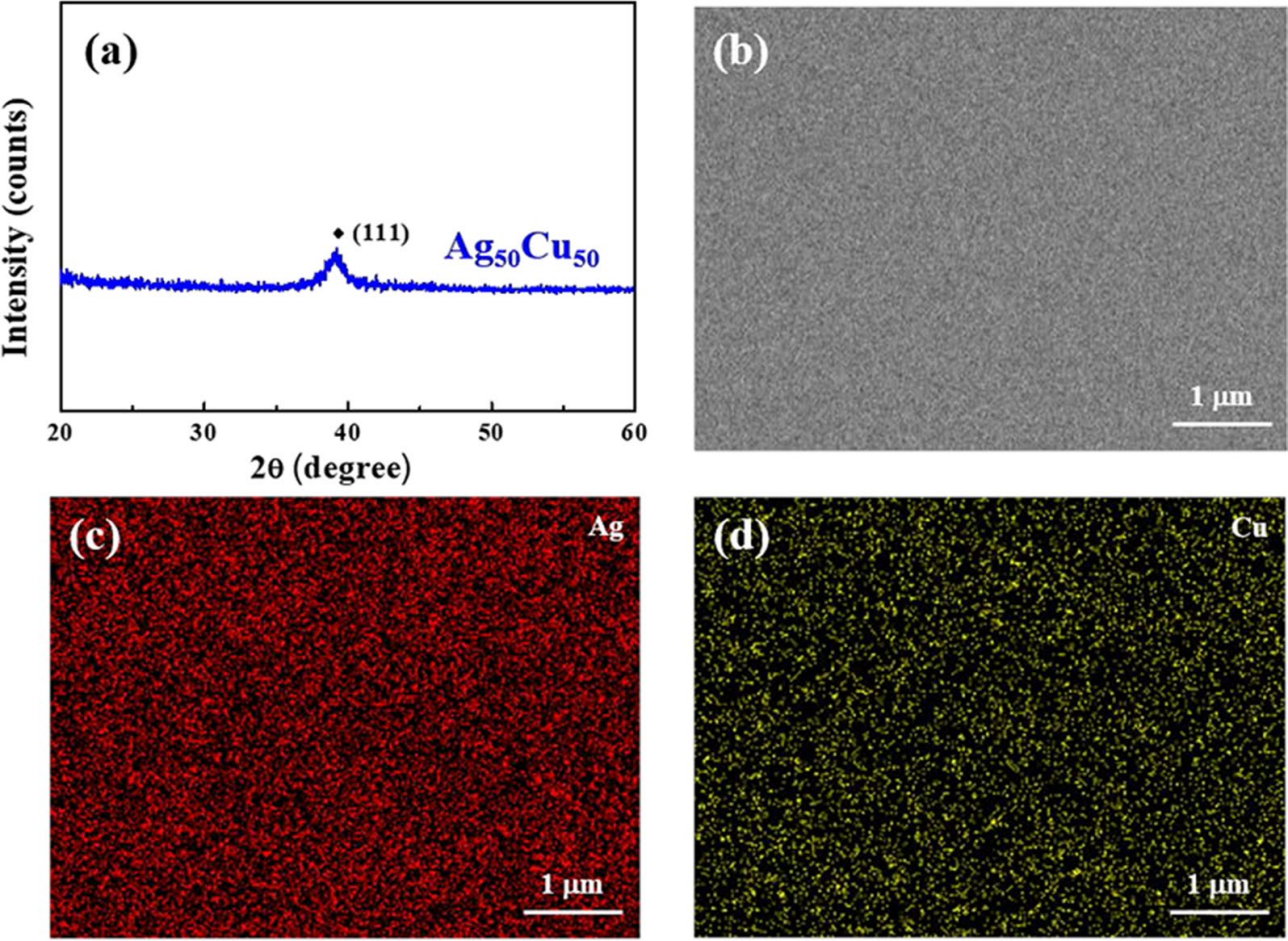
**a** XRD pattern, **b** SEM image, and the corresponding composition mapping, **c** Ag and **d** Cu, of the as-deposited Ag_50_Cu_50_ film

Figure 1a shows the X-ray diffraction pattern of the as-deposited Ag_50_Cu_50_ film. The obvious diffraction peak in the (111) plane indicates that the film has a crystal structure. Hsieh [26] also reported that as–deposited Ag_50_Cu_50_ film only has one diffraction peak. Compared with the reference, the similar XRD result could be obtained. It is known that Cu can dissolve Ag atoms only up to 4.9 at %, while Ag can dissolve up to 14.1 at % Cu. The Ag (111) shifts to the right with the increase in Cu content. Therefore, only one diffraction peak appeared in our result. Moreover, the SEM image of the Ag_50_Cu_50_ image shown in Fig. 1b shows that film has a smooth and continuous appearance. Finally, the EDS mapping results presented in Fig. 1c, d confirm the compositional homogeneity of the Ag and Cu alloying components.

**Fig. 2:**
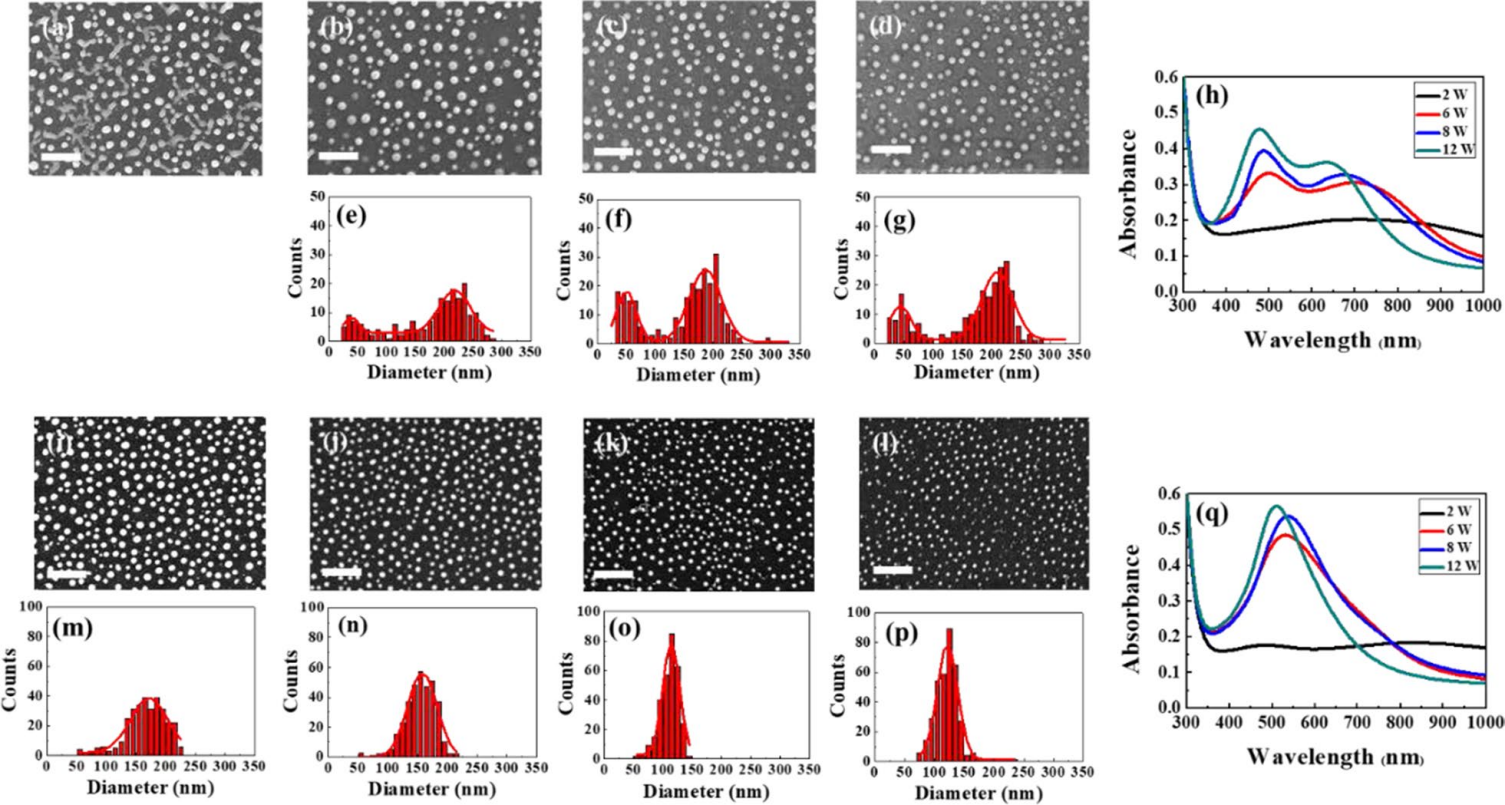
**a**–**d** Surface morphologies of dewetted nanoparticles induced using constant repetition rate (300 kHz) and scan speed (400 mm/s), but different pulse laser powers (2, 6, 8 and 12 W, respectively); **e**–**g** corresponding size distributions of nanoparticles; **h** corresponding absorption spectra. **i**–**l** Surface morphologies of dewetted nanoparticles induced using constant repetition rate (100 kHz) and scan speed (400 mm/s), but different pulse laser powers (2, 6, 8 and 12 W, respectively); **m**–**p** corresponding size distributions of nanoparticles; **q** corresponding absorption spectra. All the scale bars are equal to 1 μm

Figure 2a–h present the morphologies of the pulse-laser dewetted nanoparticles, the corresponding size distribution diagrams, and the absorption spectra of the dewetted Ag_50_Cu_50_ films produced using a constant repetition rate and scanning speed of 300 kHz and 400 mm/s, respectively, and laser powers of 2, 6, 8 and 12 W. For the processing conditions considered in Fig. 2a–h, the pulse energy varies from 6.7 to 40 μJ. Moreover, due to the high repetition rate, the accumulated energy is relatively low [13]. The size distribution plots show that the considered processing parameters result in the formation of nanoparticles with two different sizes, namely larger nanoparticles with a size of approximately 200 nm and smaller nanoparticles with a size of around 50 nm. Furthermore, the absorption spectra show the presence of two obvious peaks at around 500 and 700 nm, respectively. Notably, such a double-peak absorption spectrum has never previously been reported in laser dewetting studies.

**Fig. 3:**
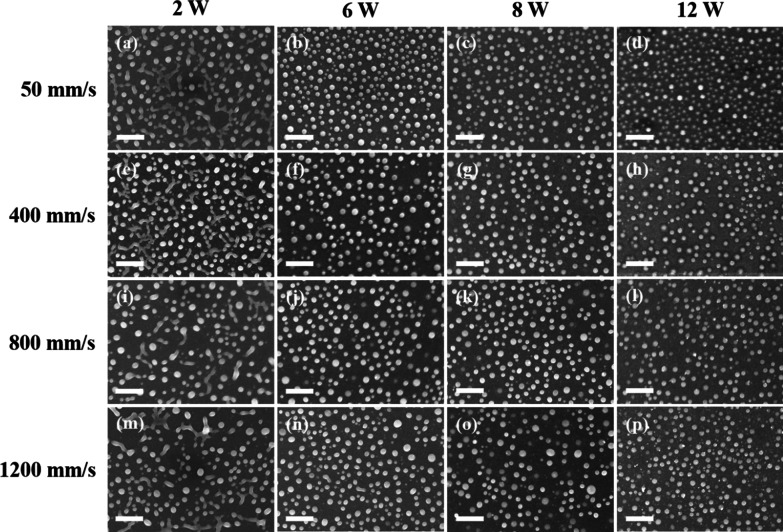
Surface morphologies of dewetted Ag_50_Cu_50_ films processed using same repetition rate (300 kHz), but different scan speeds and powers. All the scale bars are equal to 1 μm

**Fig. 4:**
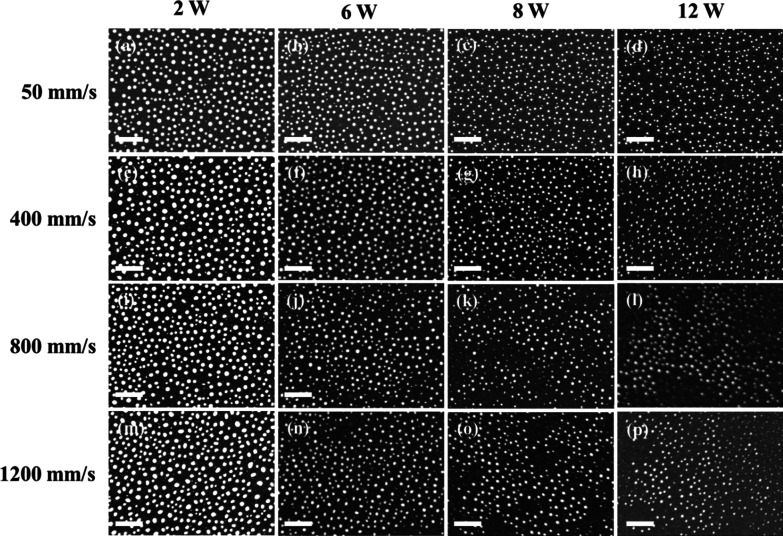
Surface morphologies of dewetted Ag_50_Cu_50_ films processed using same repetition rate (100 kHz), but different scan speeds and powers. All the scale bars are equal to 1 μm

Figure 2i–q present the morphologies, size distributions and absorption spectra of the Ag_50_Cu_50_ films processed using the same scanning speed (400 mm/s) and laser powers (2, 6, 8 and 12 W) as those described above, but a lower repetition rate of 100 kHz. In this case, the pulse energy varies from 20 to 120 μJ and the low repetition rate results in a relatively high accumulated energy [13]. It is noted that the size distributions and absorption spectra obtained under a higher accumulated energy are very different from those obtained under the lower energy condition (Fig. 2e–h). In particular, the nanoparticle size has a Gaussian distribution with a mean of 100 nm for all values of the laser power, while the absorption spectrum contains only a single peak at a wavelength of approximately 550 nm. Figures 3 and 4 show the dewetted morphologies of the Ag_50_Cu_50_ surfaces processed with different laser powers and scanning speeds at repetition rates of 300 kHz and 100 kHz, respectively. Comparing the absorption spectra shown in Fig. 2h, q, respectively, with those of pure Ag [16] and Cu [13], the absorption peaks in the two spectra lie between those of pure Ag and Cu. For the spectra shown in Fig. 2h, for a low energy accumulation, the absorption peak at around 500 nm is caused by the larger Ag_80_Cu_20_ nanoparticles, while that at the higher wavelength of 700 nm is associated with the smaller Ag_60_Cu_40_ nanoparticles. (Note that the chemical compositions of the various NPs are listed in Table 1). In other words, the higher Ag concentration results in a blue shift of the absorption peak toward a smaller wavelength. For the spectra shown in Fig. 2q, corresponding to a high energy accumulation, the single absorption peak at a wavelength of around 550 nm is also associated with nanoparticles with a composition of Ag_60_Cu_40_ (see Table 1). According to [27], the shape of nanoparticles has a significant effect on the position of the absorption peak. For example, the absorption peak of pure Ag nanoparticles with a size of 80 nm is located close to 500 nm for a spherical shape, but shifts to 650 nm for an oblate particle shape [28]. In considering the blue shift caused by a reducing particle size and the red shift caused by a higher Cu content and the shape effect, it can be concluded that the absorption peak observed in Fig. 2h at around 700 nm is the result of small Ag_60_Cu_40_ rice-shaped nanoparticles with a diameter of 50 nm. Overall, the results show that the rice shape of the smaller Ag_60_Cu_40_ nanoparticles produced in the 300-kHz sample prompts a red shift of the absorption peak from 550 to 700 nm, while the absorption peak caused by the larger hemispherical Ag_80_Cu_20_ nanoparticles remains at around 500 nm.

**Table 1:** Summed composition results at different analysis points in Figs. 5 and 9

Spectrum	Ag (at%)	Cu (at%)
1	81.1	18.9
2	80.2	19.8
3	81.6	18.4
4	58.7	41.3
5	59.5	40.5
6	58.0	42.0
7	61.0	39.0
8	60.3	39.7
9	61.8	38.2
10	62.0	38.0

**Fig. 5:**
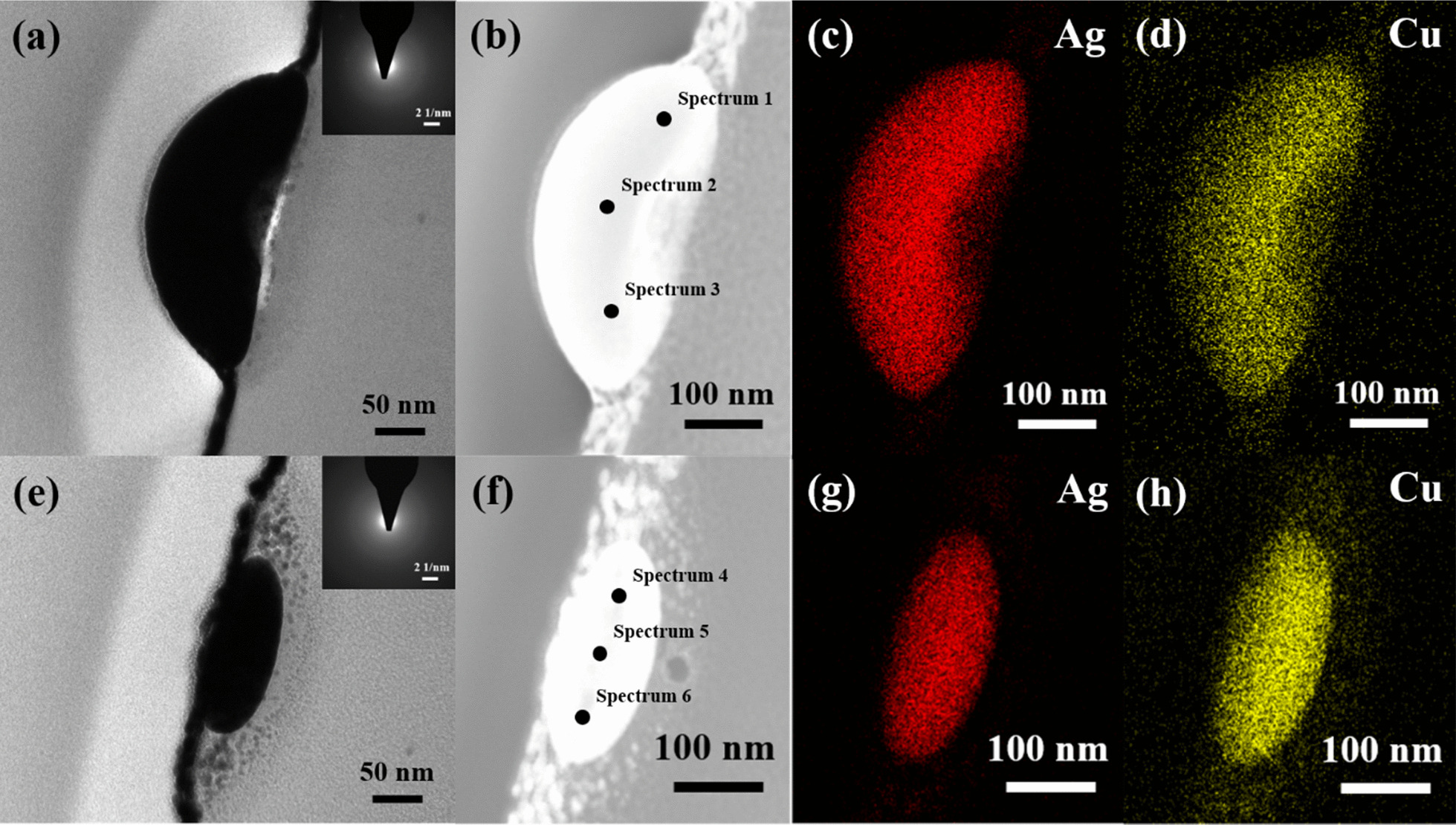
TEM analysis results for 6 W–300 kHz–400 mm/s dewetted nanoparticles. **a** Bright field image of larger nanoparticle and **b** corresponding HAADF-STEM image. EDS mapping results for **c** Ag and **d** Cu. **e** Bright field image of smaller rice-shaped nanoparticle and **f** corresponding HAADF-STEM image. EDS mapping results for **g** Ag and **h** Cu

**Fig. 6:**
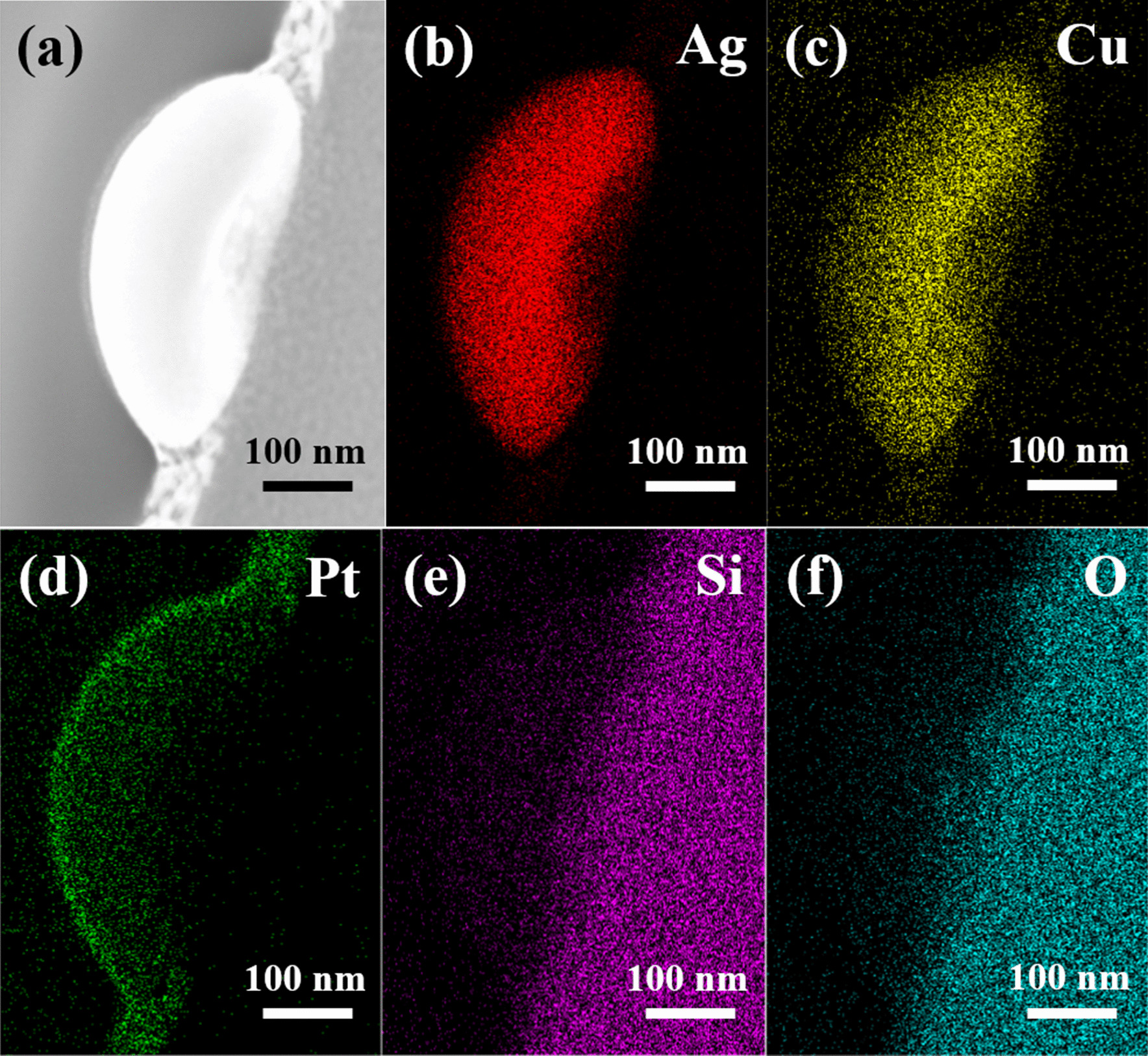
**a** HAADF STEM image of large 6 W–300 kHz–400 mm/s dewetted nanoparticle and **b**–**f** corresponding EDS mapping results. (Note that the nanoparticle has a composition of Ag_80_Cu_20_.)

**Fig. 7:**
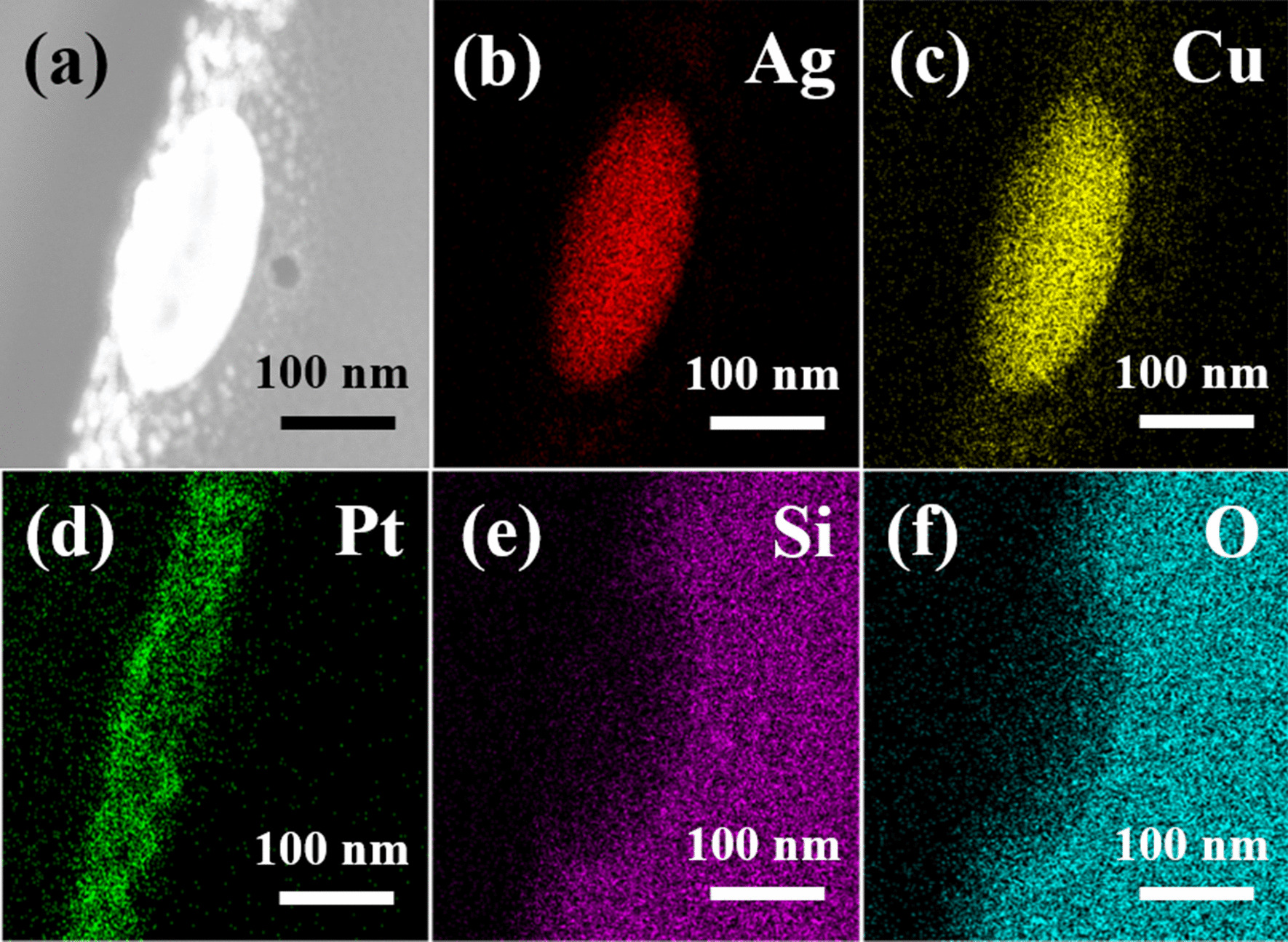
**a** HAADF STEM image of small 6 W–300 kHz–400 mm/s dewetted nanoparticle and **b**–**f** corresponding EDS mapping results. (Note that the nanoparticle has a composition of Ag_60_Cu_40_.)

A detailed cross-section TEM analysis was performed to determine the exact microstructures and element compositions of the various nanoparticles formed in the 300-kHz samples. Figure 5a, b present a bright field image and HAADF-STEM image of the large nanoparticles formed dewetted structure, respectively. The diffraction pattern shown in the inset of Fig. 5a reveals that the nanoparticle has an amorphous structure as a result of the rapid cooling rate induced in the dewetting process. A similar structure is also observed for the smaller nanoparticle produced under the same dewetting conditions (Fig. 5e). However, comparing the images shown in Fig. 5e, f) with those in Fig. 5a, b, respectively, it is seen that the smaller nanoparticles have a rice-like shape, while the larger nanoparticles have a hemispherical shape. Observing the EDS analysis results presented in Fig. 5c, d, g, h, it is found that, irrespective of the nanoparticle size, the Ag and Cu elements are evenly distributed throughout the nanoparticle structure with no obvious phase separation between them. Figures 6 and 7 show the detailed EDS mappings of the large and small nanoparticles, respectively. It is seen that both nanoparticles contain small quantities of Pt, Si and O. However, broadly speaking, the larger nanoparticle has a composition of Ag_80_Cu_20_, while the smaller nanoparticle has a composition of Ag_60_Cu_40_ (see also Table 1).

Comparing the size distributions and chemical compositions of the nanoparticles formed in the 300-kHz and 100-kHz samples, respectively, it is seen that the use of a lower repetition rate (i.e., a higher accumulated energy [13]) causes the size distribution to approach a Gaussian distribution and the nanoparticles to have a consistent Ag_60_Cu_40_ concentration. By contrast, for a higher repetition rate (i.e., a lower accumulated energy), the nanoparticles have two different sizes (50 nm and 200 nm) and two different compositions, namely Ag_60_Cu_40_ and Ag_80_Cu_20_, respectively. Interestingly, the composition of Ag_60_Cu_40_ lies at the eutectic point in the Ag–Cu binary system [29]. Overall, the results suggest that for a higher accumulated energy, the diffusion rate of the atoms is enhanced; resulting in a more even distribution of the composition elements during the dewetting process. Furthermore, it seems that the composition adjusts itself along the liquidus line and moves toward the eutectic point given the occurrence of sufficient diffusion. As a result, the whole dewetted surface is covered by Ag_60_Cu_40_ nanoparticles with a Gaussian size distribution. Moreover, the weak FCC crystalline structure observed in the nanoparticles can be attributed to the lower cooling rate associated with a higher accumulated energy. For the 300-kHz sample, the accumulated energy is reduced, which is insufficient to prompt a complete film dewetting. Thus, partial film perforation and contraction occurs; resulting in the formation of larger nanoparticles together with unstable molten metal filaments, which subsequently transform into smaller nanoparticles [30]. In other words, the larger nanoparticles experience a faster cooling rate and hence retain their original size, while the molten filaments experience a slower cooling rate and separate into smaller nanoparticles under the effects of thermal cooling. Consequently, the final dewetted film contains both large nanoparticles with a composition of Ag_80_Cu_20_ associated with a faster cooling rate and small nanoparticles with a composition of Ag_60_Cu_40_ associated with a lower cooling rate.

According to the literature [31], copper has a lower viscosity than silver. Therefore, during the dewetting process, the copper atoms diffuse faster and more readily than the silver atoms. A clear evident is the adjacent region near nanoparticles shows more Cu but fewer Ag as presented in those HAADF-STEM EDS mapping results, implying the loss of Cu “in nanoparticles”. As a result, a temporary high silver concentration (Ag_80_Cu_20_) region is formed within the nanoparticle. Note that the role of diffusion (rather than evaporation) in prompting a loss of Cu within the nanoparticles is supported by the relatively higher boiling temperature of Cu (2562 °C) than Ag (2162 °C), which suggests that the Cu loss is unlikely to be the result of evaporation. Nonetheless, despite the generally low diffusion rate, some regions of the dewetted film still experience sufficient diffusion, and thus small rice-shaped nanoparticles with a composition of Ag_60_Cu_40_ are formed.

**Fig. 8:**
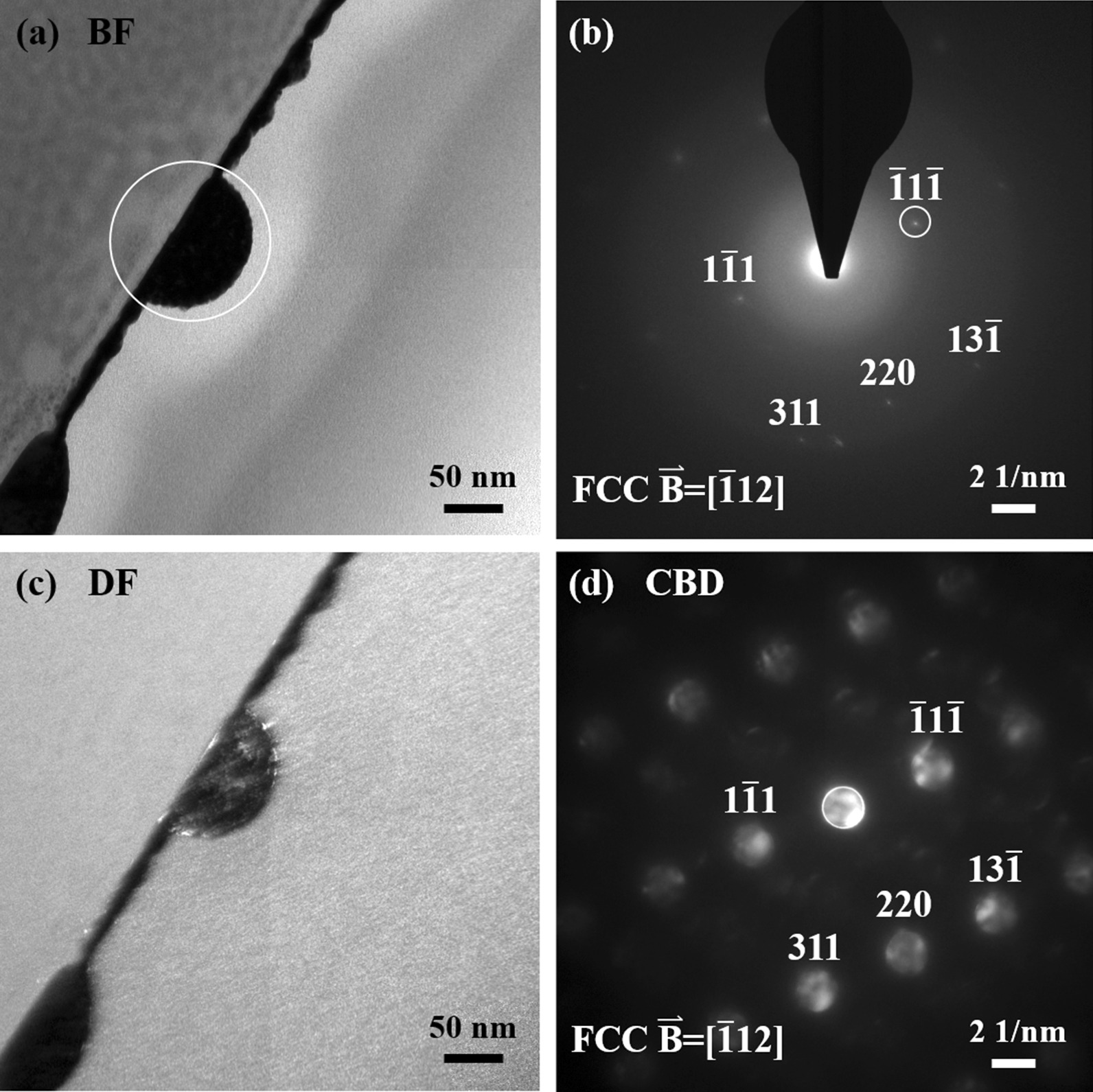
**a** Bright field image and corresponding **b** diffraction pattern and **c** dark field image of 6 W–100 kHz–400 mm/s dewetted nanoparticle. **d** Convergent beam diffraction pattern showing FCC structure

**Fig. 9:**
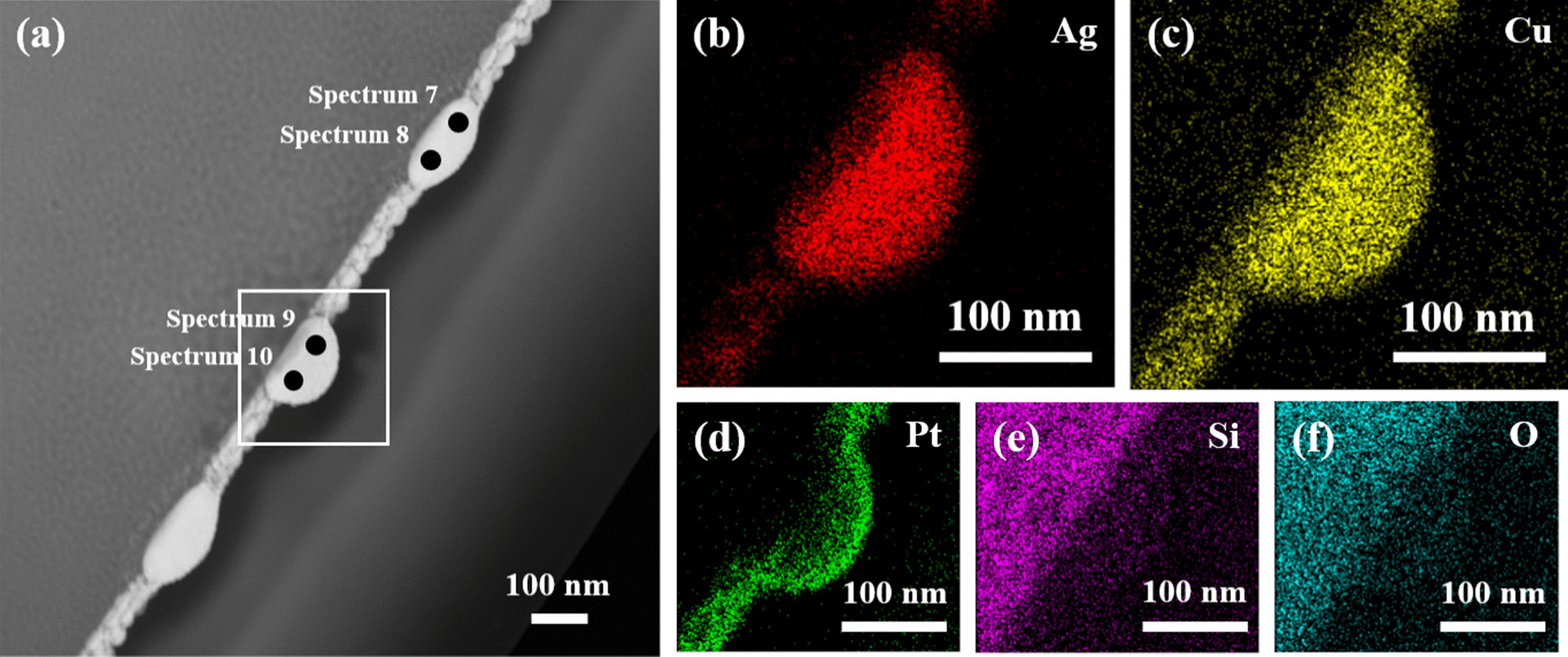
**a** HAADF STEM image of 6 W–100 kHz–400 mm/s dewetted nanoparticles and **b**–**f** corresponding EDS mapping results

Figure 8 shows the cross-section TEM analysis results for the nanoparticles in the 100-kHz sample. The bright field image presented in Fig. 8a shows that the nanoparticles also have a hemispherical shape. However, the diffraction pattern in Fig. 8b shows that they have an FCC structure. Nonetheless, most of the regions in the nanoparticle have an amorphous microstructure. As described above, this can be attributed to the rapid cooling rate during the dewetting process. However, the cooling rate for the film processed with a repetition rate of 100 kHz is lower than that for the film processed with a repetition rate of 300 kHz, and hence the nanoparticles have a weak crystalline structure, as evidenced by a comparison of the diffraction pattern in Fig. 8b with that in the inset of Fig. 5a. Nonetheless, the convergent beam diffraction image shown in Fig. 8d confirms that the nanoparticles in the 100-kHz sample have an FCC structure. The HAADF-STEM image (Fig. 9a) and corresponding EDS mapping results (Fig. 9b–f) show that the Ag and Cu elements are uniformly distributed throughout the hemispherical nanoparticles without any significant phase separation. Moreover, the composition of the nanoparticles is approximately Ag_60_Cu_40_, as shown in Table 1.

**Fig. 10:**
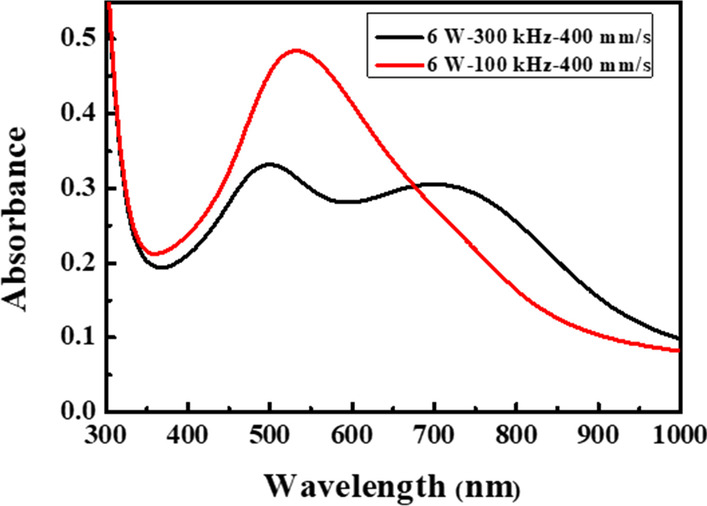
Wider examination of absorption spectrum from 300 to 1000 nm in 300-kHz and 100-kHz samples

It is theoretically possible that the two absorption peaks in the spectra of the 300-kHz samples are the result of both dipole and quadrupole plasmon resonance, as reported previously in the literature for large nanoparticles with a size of 140 nm [28]. Thus, Fig. 10 presents a wider examination (300–1000 nm) of the absorption spectra in the 300-kHz and 100-kHz samples. It is noted that the absorption peak characteristic of Ag quadrupole plasmon resonance at 300–400 nm is absent in both spectra. Since the nanoparticles in both samples are sufficiently large to support quadrupole plasmon resonance [32], the absence of such a peak implies that the double peak absorption spectrum observed for the 300-kHz samples is the result of size distribution, nanoparticle shape and nanoparticle composition effects rather than quadrupole plasmon resonance.

## Conclusions

This study has investigated the effect of the accumulated energy induced by different repetition rates on the dewetted morphologies of Ag_50_Cu_50_ thin films. The results have shown that given the use of a lower repetition rate, the diffusion rate of the atoms during the dewetting process increases. The resulting nanoparticles have an even composition distribution of Ag_60_Cu_40_, a mean diameter of 100 nm and a peak absorbance wavelength of 550 nm. By contrast, for a higher repetition rate, the diffusion rate of the atoms is suppressed. Thus, the dewetted structure contains two different types of nanoparticles, namely large hemispherical nanoparticles with a composition of Ag_80_Cu_20_ and small rice-shaped nanoparticles with a composition of Ag_60_Cu_40_. The corresponding absorption spectrum contains two peaks at wavelengths of 500 nm and 700 nm, respectively. It is thus speculated that a higher concentration of Ag in the nanoparticles results in a blue shift of the peak in the absorption spectrum, while a rice shape of the nanoparticles causes a red shift of the peak in the absorption spectrum.

## Data Availability

The datasets used and/or analysed during the current study are available from the corresponding author on reasonable request.

## Abbreviations

LSPR: Localized surface plasmon resonance
XRD: X-ray diffraction
NIR: Near-infrared radiation
FE-SEM: Field-emission scanning electron microscope
FE-TEM: Field-emission transmission electron microscope
EDS: Energy dispersive x-ray spectrometry
FIB: Focus ion beam
FCC: Face centered cubic

## References

[1] Shim K, Lin J, Park M-S, Shahabuddin M, Yamauchi Y, Hossain MSA, Kim JH (2019). Tunable porosity in bimetallic core-shell structured palladium-platinum nanoparticles for electrocatalysts. Scr Mater.

[2] Marvel C, Kracum M, Yu Z, Harmer M, Chan H (2018). Observation of Cu-rich grain boundary nanoparticles and complexions in Cu/Ti-doped alumina. Scr Mater.

[3] Huerta-Murillo D, Aguilar-Morales AI, Alamri S, Cardoso JT, Jagdheesh R, Lasagni AF, Ocaña JL (2017). Fabrication of multi-scale periodic surface structures on Ti-6Al-4V by direct laser writing and direct laser interference patterning for modified wettability applications. Opt Lasers Eng.

[4] Rycenga M, Cobley CM, Zeng J, Li W, Moran CH, Zhang Q, Qin D, Xia Y (2011). Controlling the synthesis and assembly of silver nanostructures for plasmonic applications. Chem Rev.

[5] Haes AJ, Van Duyne RP (2004). A unified view of propagating and localized surface plasmon resonance biosensors. Anal Bioanal Chem.

[6] Zuo Z, Zhu K, Wen Y, Zhang S (2018). Quadrupolar plasmon resonance in arrays composed of small-sized Ag nanoparticles prepared by a dewetting method. Appl Surf Sci.

[7] Eremenko A, Petrik I, Smirnova N, Rudenko A, Marikvas Y (2016). Antibacterial and antimycotic activity of cotton fabrics, impregnated with silver and binary silver/copper nanoparticles. Nanoscale Res Lett.

[8] Lin YH, Wang JJ, Wang YT, Lin HK, Lin YJ (2020). Antifungal properties of pure silver films with nanoparticles induced by pulsed-laser dewetting process. Appl Sci.

[9] Wongrat E, Wongkrajang S, Chuejetton A, Bhoomanee C, Choopun S (2019). Rapid synthesis of Au, Ag and Cu nanoparticles by DC arc-discharge for efficiency enhancement in polymer solar cells. Mater Res Innov.

[10] Huang CL, Kumar G, Sharma GD, Chen FC (2020). Plasmonic effects of copper nanoparticles in polymer photovoltaic devices for outdoor and indoor applications. Appl Phys Lett.

[11] Kwon J, Suh YD, Lee J, Lee P, Han S, Hong S, Yeo J, Lee H, Ko SH (2018). Recent progress in silver nanowire based flexible/wearable optoelectronics. J Mater Chem C.

[12] Kushwah M, Bhadauria S, Arora K, Gaur M (2019). Enhanced catalytic activity of chemically synthesized Au/Ag/Cu trimetallic nanoparticles. Mater Res Express.

[13] Lin H, Wang Y, Chuang W, Chou H, Huang J (2020). Surface resonance properties of pure Cu and Cu80Zr20 metallic glass films with nanoparticles induced by pulsed-laser dewetting process. Appl Surf Sci.

[14] Oh H, Pyatenko A, Lee M (2019). Laser dewetting behaviors of Ag and Au thin films on glass and Si substrates: experiments and theoretical considerations. Appl Surf Sci.

[15] Oh Y, Lee J, Lee M (2018). Fabrication of Ag–Au bimetallic nanoparticles by laser-induced dewetting of bilayer films. Appl Surf Sci.

[16] Oh Y, Lee M (2017). Single-pulse transformation of Ag thin film into nanoparticles via laser-induced dewetting. Appl Surf Sci.

[17] Ruffino F, Grimaldi MG (2019). Nanostructuration of thin metal films by pulsed laser irradiations: a review. Nanomaterials.

[18] Henley SJ, Carey JD, Silva SRP (2007). Metal nanoparticle production by pulsed laser nanostructuring of thin metal films. Appl Surf Sci.

[19] Chen K, Yuan D, Zhao Y (2021). Review of optical hydrogen sensors based on metal hydrides: recent developments and challenges. Opt Laser Technol.

[20] Klar T, Perner M, Grosse S, von Plessen G, Spirkl W, Feldmann J (1998). Surface-plasmon resonances in single metallic nanoparticles. Phys Rev Lett.

[21] Abalde-Cela S, Carregal-Romero S, Coelho JP, Guerrero-Martinez A (2016). Recent progress on colloidal metal nanoparticles as signal enhancers in nanosensing. Adv Colloids Interface Sci.

[22] Ansari Z, Saha A, Singha SS, Sen K (2018). Phytomediated generation of Ag, CuO and Ag-Cu nanoparticles for dimethoate sensing. J Photochem Photobiol A.

[23] Ruffino F, Grimaldi MG (2018). Roughness evolution in dewetted Ag and Pt nanoscale films. Superlattices Microstruct.

[24] Zaleska-Medynska A, Marchelek M, Diak M, Grabowska E (2016). Noble metal-based bimetallic nanoparticles: the effect of the structure on the optical, catalytic and photocatalytic properties. Adv Colloids Interface Sci.

[25] Benware BR, Macchietto CD, Moreno CH, Rocca JJ (1998). Demonstration of a high average power tabletop soft X-Ray Laser. Phys Rev Lett.

[26] Hsieh J, Hung S. The effect of Cu:Ag atomic ratio on the properties of sputtered Cu-Ag alloy thin films. Materials (Basel) 9(11) (2016). 10.3390/ma9110914 PMC5457197 28774033

[27] Wiley BJ, Chen Y, McLellan JM, Xiong Y, Li Z-Y, Ginger D, Xia Y (2007). Synthesis and optical properties of silver nanobars and nanorice. Nano Lett.

[28] Kelly KL, Coronado E, Zhao LL, Schatz GC (2003). The optical properties of metal nanoparticles: the influence of size, shape, and dielectric environment. J Phys Chem B.

[29] Wu J, Lee CC (2018). Low-pressure solid-state bonding technology using fine-grained silver foils for high-temperature electronics. J Mater Sci.

[30] Henley SJ, Poa CHP, Adikaari AADT, Giusca CE, Carey JD, Silva SRP (2004). Excimer laser nanostructuring of nickel thin films for the catalytic growth of carbon nanotubes. Appl Phys Lett.

[31] Egry I, Lohöfer G, Sauerland S (1993). Surface tension and viscosity of liquid metals. J Non-Cryst Solids.

[32] Kelly KL, Coronado E, Zhao LL, Schatz GC (2003). The optical properties of metal nanoparticles: the influence of size, shape, and dielectric environment. J Phys Chem B.

